# Bioactive Compounds from Mangrove Endophytic Fungus and Their Uses for Microorganism Control

**DOI:** 10.3390/jof7060455

**Published:** 2021-06-07

**Authors:** Rafael Dorighello Cadamuro, Isabela Maria Agustini da Silveira Bastos, Izabella Thais Silva, Ariadne Cristiane Cabral da Cruz, Diogo Robl, Louis Pergaud Sandjo, Sergio Alves, Jose M. Lorenzo, David Rodríguez-Lázaro, Helen Treichel, Mário Steindel, Gislaine Fongaro

**Affiliations:** 1 Department of Microbiology, Immunology, and Parasitology, Federal University of Santa Catarina, Florianópolis 88040-900, SC, Brazil; rafaelcada@hotmail.com (R.D.C.); isabelamaria646@gmail.com (I.M.A.d.S.B.); izabella.thais@ufsc.br (I.T.S.); ariadne.cruz@ufsc.br (A.C.C.d.C.); diogo.robl@ufsc.br (D.R.); msteindel@gmail.com (M.S.); 2 Department of Pharmaceutical Sciences, Federal University Santa Catarina, Florianopolis 88040-900, SC, Brazil; 3 Department of Dentistry, Federal University of Santa Catarina, Florianópolis 88040-900, SC, Brazil; 4 Department of Chemistry, Federal University of Santa Catarina, Florianópolis 88040-900, SC, Brazil; p.l.sandjo@ufsc.br; 5 Laboratory of Biochemistry and Genetics, Federal University of Fronteira Sul, Chapecó 89802-112, SC, Brazil; slalvesjr@gmail.com; 6 Centro Tecnológico de la Carne de Galicia, Avd. Galicia n° 4, Parque Tecnológico de Galicia, San Cibrao das Viñas, 32900 Ourense, Spain; 7 Área de Tecnología de los Alimentos, Facultad de Ciencias de Ourense, Universidad de Vigo, 32004 Ourense, Spain; 8 Microbiology Division, Faculty of Sciences, University of Burgos, 09001 Burgos, Spain; drlazaro@ubu.es; 9 Laboratory of Microbiology and Bioprocess, Federal University of Fronteira Sul, Erechim 99700-000, RS, Brazil; helentreichel@gmail.com

**Keywords:** biotechnology, biodiversity, new drugs, health, pathogen control

## Abstract

Mangroves are ecosystems with unique characteristics due to the high salinity and amount of organic matter that house a rich biodiversity. Fungi have aroused much interest as they are an important natural source for the discovery of new bioactive compounds, with potential biotechnological and pharmacological interest. This review aims to highlight endophytic fungi isolated from mangrove plant species and the isolated bioactive compounds and their bioactivity against protozoa, bacteria and pathogenic viruses. Knowledge about this type of ecosystem is of great relevance for its preservation and as a source of new molecules for the control of pathogens that may be of importance for human, animal and environmental health.

## 1. Introduction

The extensive and continued use of natural products in popular medicine may be considered an indicator that they contain bioactive molecules with the potential to be transformed into new therapeutic agents for use in the treatment of diseases [1]. There are many examples of medicines (antibiotics, antiviral, anti-fungal, anti-parasitic, anti-tumoral, anticholesterolemic, anti-hypertensive, among others) from natural products, notably from higher plants, microorganisms and animals, among the best-sellers worldwide. According to Cragg and Newman [2,3], from the 1562 drugs approved by the FDA between 1981 and 2014, around 525 (33.7%) were natural products or natural product derivatives. The use of natural products in the drug discovery process and development has some clear advantages: they represent chemical novelties when compared with other sources, leading to new drug candidates for complex targets [4,5]. By contrast, access to natural biological resources by lack of government legislation sometimes makes it challenging to use naturally derived molecules as a source of new medicines. However, naturally derived constituents have an extraordinary chemical diversity, compared to any collection of synthetic chemicals, and despite having differences such as complex two-dimensional and three-dimensional structures, pharmacological target, selectivity, behavior and resistance, they are capable of being absorbed and metabolized in the body [6].

The search for new bioactive compounds is three-fold: (i) find molecules that may control diseases that no synthetic drug has been shown to be capable of, (ii) discover alternative compounds that provoke fewer side-effects and lower multi-drug resistance over the microbiomes and (iii) replace synthetic drugs to mitigate environmental impacts caused by their presence in soil and bodies of water [6,7,8]. Natural products obtained from microorganisms (microbial products) are generally used for the treatment of diseases caused by bacteria, fungi, protozoa and viruses. Microorganisms have stood out in the production of new natural products. Out of the 23,000 existing microbial compounds with antimicrobial and anti-infectious activities, 42% are produced by fungi and 32% by filamentous bacteria, the actinomycetes [9]. 

The production of antibiotics began with the discovery of penicillin at the end of the 1920s [10,11]. After the 1980s, pharmaceutical companies began to lose interest in the development of new compounds, as each new discovery takes years of development, requires both pre-clinical and clinical studies and has a short window of time for organizations to sell the products before the expiration of the patent. The most intensive use of classical antibiotics occurred in the era of antibiotics (1940–1962), so new antibiotics are necessary for the treatment of diseases since pathogens in their great majority can create resistance to old natural products, while some have this resistance naturally, such as *Pseudomonas aeruginosa* [9]. The search for new compounds for drug production is challenging. The screening of new compounds requires a lot of knowledge, scientific experience and the use of technology [12].

The mangrove ecosystem is an attractive biodiversity hotspot for prospecting new useful bioactive and chemical scaffolds, including those with potential medicinal application. Overall, in the past two decades, mangrove-associated bacteria/fungi have gained considerable attention due to their unique ecological characteristics, diversity and abundance of novel bioactive secondary metabolites, as demonstrated by the growth in the number of publications in the literature [13,14]. Mangrove forests are composed mainly of shrubs and trees of the *Rhizophoraceae, Acanthaceae, Lythraceae, Verbenaceae, Combretaceae* and *Arecaceae* families [14]. In Brazil, mangroves are classified as red, white or black mangroves according to some of these families: *Rhizophora mangle* (*Rhizophoraceae*) [15], *Laguncularia racemosa* (*Combretaceae*) [15] and *Avicennia schaueriana* (*Verbenaceae*) [16], respectively. These species can be found mainly in the city of Florianópolis, which is the capital city of the state of Santa Catarina, and is located on an island, together with the species *Spartina densiflora* and *Spartina alterniflora* weeds. In the transitional forest area, there are also species such as *Hibiscus pernambucensis* and *Acrostichum danaeaefolium*, commonly known as “mangrove cotton” and “mangrove fern” [17].

The endemic mangrove flora represents a great source of molecules with biological potential produced by plant biosynthesis, microbial interaction and cohabitation with other species [18]. Beyond this, mangroves have fauna rich in aquatic animals (fish, amphibians and reptiles) and land animals (mammals and birds). These animals take advantage of mangrove forests for their essential life cycle activities, and their breeding and reproducing cycles provide a rich source of food for humans [19].

Mangrove forest is a refuge for several microorganisms, such as fungi, bacteria and algae. In this review, we focused on endophytic fungi from mangrove ecosystems as a potential source of new natural products with biotechnological and pharmaceutical applications. Moreover, methodologies used for the isolation of these microorganisms are also presented.

Endophytic fungi isolated from mangrove plants and mangrove soils were first described by Cribb [20]. Since then, several studies on these marine fungi have been conducted along the coast of the Indian, Pacific and Atlantic oceans. These fungi comprise the second-largest ecological group of marine fungi. They have unique morphological structures and physiological mechanisms for the survival of host plants in adverse environmental conditions, such as the ability to grow in high salt concentrations through endophyte–host interactions [21].

Many of the physiological mechanisms of endophytic–host interaction are still poorly understood and established, mainly those related to the evolutionary and genetic mechanisms of the endophytes. It is supposed that the endophytic species have often evolved from plant pathogenic ancestors, and that this interaction can range from parasitism to mutualism, which depends mainly on the fungi species, the genetic background of the host and the environment where these microorganisms are found [22].

## 2. Endophytic Fungi

Endophytic fungi are phylogenetically characterized as belonging to the *Ascomycota*, *Basidiomycota* and *Zygomycota* phyla [23]. The ascomycetes of the genus Trichoderma, reported in the literature in the last ten years, were first isolated from mangrove areas of Brazil, China and Indonesia in 1920 (Table 1). Known as a biocontrol agent against pathogens of cultivated plants, *Trichoderma* spp. also present an increased capacity of degradation of some toxic compounds present in plants, soil and water [24]. *Trichoderma* spp. colonizes its hosts quickly, producing a large number of green spores of free life, and has fruiting bodies that assist in the fungal characterization of this genus [25]. 

Zygomycetes of *Rhizopus* genera isolated in a mangrove area of Nigeria (Table 1) is also a filamentous fungus that presents branched mycelium bodies. It is mainly used in traditional food fermentation processes and as a source of enzymes for degradation of organic pollutants [26,27]. The filamentous fungi *Schizophyllum commune*, isolated from the Indian mangrove forest (Table 1), belongs to the *basidiomycete* phylum. It has fruiting bodies (which facilitate its characterization) and whitish to light greyish/brown colonies. It is used in pigment production and has antiviral and anticancer capacities [28]. 

The diversity of endophytic fungi has been studied mainly on barks, branches, leaves, stems and roots of mangrove plant taxa in many countries around the world in order to identify their biological activities. It is noteworthy that China is the country with the highest number of endophytic fungi isolated from mangrove plants. Ascomycetes, belonging to the *Alternaria, Ascomycota, Aspergillus, Campylocarpon, Cladosporium, Colletotrichum, Cytospora, Daldinia, Diaporthe, Dothiorella, Emericella, Eupenicillium, Eurotium, Guignardia, Glomerella, Lasiodiplodia, Leptosphaerulina, Neosartorya, Nodulisporium, Nigrospora, Penicillium, Pestalotiopsis, Phoma, Phomopsis, Phyllosticta, Pleosporales, Stemphylium, Talaromyces, Trichoderma* and *Xylaria* genera, and the basidiomycete *Phellinus noxius,* were the most frequently found (Table 1).

In Brazil, endophytic ascomycetes of the *Colletotrichum, Glomerella, Guignardia, Nodulisporium, Phomopsis* and *Phyllosticta* genera were isolated in a mangrove area of the island of Itamaracá in the state of Pernambuco [29]. Isolations have also occurred in the Cananeia and Bertioga mangrove forests in the coast of the state of São Paulo, with the predominance of ascomycetes of the *Colletotrichum, Diaporthe, Fusarium, Trichoderma* and *Xylaria* genera [30]. More recently, ascomycetes from the *Aspergillus, Fusarium, Penicillium* and *Trichoderma* genera have been isolated from a mangrove area in the city of Canavieiras, in the state of Bahia [31]. Thus, considering the vast coastal extension of Brazil with different ecosystems, with a distance between Bahia and Santa Catarina higher than 1.900 km, for example, this way, there are few studies of endophytic fungi from mangrove plants.

**Table 1:** Endophytic fungi isolated from mangrove plants worldwide.

Endophytic Fungi	Mangrove Plant	Reference
***Acremonium* sp. and *Acremonium strictum***	*Rhizophora apiculata*	[32,33]
***Alternaria longipe***	*Avicennia officinalis*	[34]
***Alternaria* sp.**	*Myoporum bontioides, Rhizophora mucronata*	[35,36]
***Ascomycota* sp.**	*Pluchea indica*	[37]
***Aspergillus clavatus***	*Myoporum bontioides*	[38]
***Aspergillus flavipes***	*Acanthus ilicifolius*	[39]
***Aspergillus flavus***	*Hibiscus tiliaceus, Sonneratia griffithii, Kandelia obovata*	[40,41,42]
***Aspergillus fumigatus***	*Acrostichum specioum, Sonneratia griffithii*	[41,43]
***Aspergillus nidulans***	*Rhizophora stylosa*	[44,45]
***Aspergillus niger***	*Sonneratia apetala,S. griffithii*	[41,46,47,48]
***Aspergillus* sp.**	*Bruguiera gymnorrhiza, Avicennia africana, Xylocarpus moluccensis, Acanthus ilicifolius, Avicennia marina, Dalbergia ecastaphyllum*	[31,49,50,51,52,53]
***Aspergillus tubingensis***	*Pongamia pinnata*	[54]
***Aspergillus versicolor***	*Excoecaria agallocha*	[55]
***Campylocarpon* sp.**	*Sonneratia caseolaris*	[56]
***Cladosporium* sp.**	*Rhizophora apiculata, Aegiceras corniculatum, Kandelia candel, Rhizophora mucronata, Excoecaria agallocha*	[36,52,57,58,59]
***Colletotrichum gloeosporioides***	*Avicennia schaueriana and Laguncularia racemosa, Ceriops tagal* and *Sonneratia apetala*	[29,47,60]
***Colletotrichum* sp.**	*Xylocarpus granatum, Avicennia schaueriana Laguncularia racemosa* and *Rhizophora mangle Aegiceras corniculatum, Avicennia africana,*	[29,50,57]
***Cytospora* sp.**	*Ceriops tagal*	[61]
***Daldinia eschscholtzii***	*Bruguiera sexangula var. rhynchopetala*	[62]
***Diaporthe* sp.**	*Avicennia schaueriana, Laguncularia racemosa,* and *Rhizophora mangle, Rhizophora stylosa*	[30,63]
***Dothiorella* sp.**	*Aegiceras corniculatum*	[64]
***Emericella* sp.**	*Aegiceras corniculatum*	[65]
***Epicoccum* sp.**	*Avicennia africana*	[50]
***Eupenicillium* sp.**	*Xylocarpus granatum*	[66]
***Eurotium chevalier***	*Rhizophora mucronata*	[67]
***Eurotium rubrum***	*Hibiscus tiliaceu*	[68]
***Fusarium equiseti***	*Sonneratia apetala*	[47]
***Fusarium lateritium***	*Rhizophora mucronata*	[36]
***Fusarium napiforme***	*Rhizophora mucronata*	[69]
***Fusarium phyllophilum***	*Avicennia africana*	[50]
***Fusarium* sp.**	*Avicennia schaueriana, Laguncularia racemosa, Rhizophora mangle, Rhizophora mucronata,* and *Dalbergia ecastaphyllum*	[30,36,69]
***Glomerella cingulata* and *Guignardia* sp.**	*Avicennia schaueriana, Laguncularia racemosa, Rhizophora mangle*	[29]
***Guignardia camelliae***	*Avicennia* sp.	[70]
***Guignardia* sp.**	*Scyphiphora hydrophyllacea, Aegiceras corniculatum, Acanthus ilicifolius;*	[59,71,72]
***Glomerella* sp.**	*Aegiceras corniculatum*	[59]
***Hypocrea virens***	*Premna serratifolia*	[73]
***Lasiodiplodia theobromae***	*Acanthus ilicifolius*, *Avicennia lanata*	[74,75]
***Leptosphaerulina* sp.**	*Acanthus ilicifolius*	[76]
***Neosartorya hiratsukae***	*Avicennia* sp. and *Aricennia marina*	[76,77]
***Nodulisporium gregarium***	*Avicennia schaueriana*	[29]
***Nodulisporium* sp.**	*Acanthus ilicifolius*	[72]
***Nigrospora* sp.**	*Kandelia candel, Pongamia pinnata,* and *Rhizophora mucronata*	[36,78,79]
***Nigrospora sphaerica***	*Bruguiera gymnorrhyza*	[79]
***Phellinus noxius***	*Acanthus ilicifolius*	[72]
***Penicillium brocae***	*Avicennia marina*	[80,81]
***Penicillium chrysogenum***	*Porteresia coarctata, Myoporum bontioides*	[82,83]
***Penicillium citrinum***	*Bruguiera sexangula* var. *rhynchopetala*	[84,85]
***Penicillium coffeae* and *Penicillium herquei***	*Laguncularia racemosa*	[86,87]
***Penicillium simplicissimum***	*Bruguiera sexangula var. rhynchopetala*	[88]
***Penicillium* spp.**	*Bruguiera sexangula var. Rhynchopetala, Bruguiera gymnorrhiza, K. candel, Avicennia africana, Dalbergia ecastaphyllum*	[31,50,89,90,91]
***Pestalotiopsis* sp.**	*Aegiceras corniculatum, Rhizophora mucronata, Rhizophora stylosa*	[36,92,93,94]
***Pestalotiopsis vacinii***	*Kandelia candel*	[7]
***Phoma* sp.**	*Thespesia populneoide, Myoporum bontioides, Rhizophora mucronata, Kandelia* sp., *Acanthus ilicifolius*	[36,38,72,95]
***Phomopsis archeri, P. diachenii***	*Avicennia schaueriana* and *Laguncularia racemosa*	[29]
***Phomopsis longicolla***	*Brguiera sexangula var. rhynchopetala*	[38]
***Phomopsis* sp.**	*Rhizophora apiculata, Kandelia candel, Acanthus ilicifolius, Xylocarpus granatum, Avicennia africana*	[50,96,97,98]
***Phyllosticta capitalensis***	*Bruguiera sexangula*	[94]
***Phyllosticta* sp.**	*Acanthus ilicifolius, Avicennia alba, Ceriops decandra, Lumnitzera littorea, Rhizophora apiculata, Rhizophora mucronata, Sonneratia alba, Xylocarpus moluccensis, Rhizophora mangle*	[28,57]
***Pleosporales* sp.**	*Kandelia candel*	[99]
***Rhizopus* sp.**	*Avicennia africana*	[50]
***Schizophyllum commune***	*Avicennia officinalis*	[100]
***Stemphylium* sp.**	*Bruguiera sexangula* var. *rhynchopetala*	[101,102]
***Talaromyces* sp.**	*Kandelia candel*	[103]
***Talaromyces stipitatus***	*Acanthus ilicifolius*	[98]
***Trichoderma* sp.**	*Avicennia schaueriana Laguncularia racemosa, Rhizophora mangle, Clerodendrum inerme, Ceriops tagal, Bruguiera* sp., *Dalbergia ecastaphyllum*	[30,70,103,104]
***Xylaria psidii***	*Aegle marmelos*	[104]
***Xylaria* sp.**	*Avicennia schaueriana, Laguncularia racemosa*, *Rhizophora mangle, Rhizophora mucronata Xylocarpus granatum, Acanthus ilicifolius*	[30,36,51,72]
***Zasmidium* sp.**	*L. racemosa*	[105,106]

## 3. Bioactive Compounds from Mangrove Endophytic Fungus

The main classes of endophytic fungal compounds isolated from mangrove areas and their pharmacological activities are presented in Table 2. The potential biological applications of metabolites produced by these fungi include growth inhibition of bacteria and protozoan and virus inactivation [106]. 

Saad [105] isolated endophytic fungi from root samples of Malva parviflora and leaf samples of *Chenopodium album, Pelargonium graveolens* and *Melia azedarach*. Nine fungi presented bioactivity and were identified using DNA-sequences, with five being isolated from C. album: *Fusarium chlamydosporum, A. alternata* saad5 MG786542, *A. alternata* saad8 MG786545, *Fusarium oxysporum* and *Phoma* sp. Two fungi were isolated from *M. azedarach*: *F. equiseti* and *Stemphylium* sp., and two from the medicinal plant *M. azedarach: C. lunata* and *Nigrospora sphaerica*. The metabolites presented bioactivity against *Spodoptera littoralis*, a lepidopteran pest known to infect around 44 different families of hosts, such as cruciferous, legumes, grasses and deciduous fruit trees. Metabolites produced by the fungi *Curvularia lunata* and *Alternaria solani* demonstrated bioactivity, inhibiting 60% and 40% of larvae of *Spodoptera littoralis*, respectively.

It is expected that some of the endophytic-fungi extracts harbor bioactive compounds when the fungal cells have been grown in the presence of epigenetic regulators, which are able to modulate gene expression for secondary-metabolite synthesis [48,107,108,109,110,111,112,113,114,115]. Recently, Demers [116] showed that 72% of the analyzed mangrove fungi presented active extracts only when cultured in media containing histone deacetylase inhibitors (HDACi) and DNA methyltransferase inhibitors (DNMTi). In addition, those authors also showed that nearly 70% of the active extracts were selective to a single target organism. Thus, considering the specificity of each endophytic-fungi bioactive against different microorganisms, the effects of these compounds against protozoan, bacteria and virus are addressed below in separate subsections.

### 3.1. Antiprotozoan

Malaria is an example of a tropical disease caused by *Plasmodium* spp., which accounts for around 220 million cases of the disease and 435,000 deaths worldwide every year [87]. The emergence of strains of malaria resistant to synthetic classical drugs requires a continuous search for new compounds from alternative niches to introduce new and efficient products to the treatment [107].

The compound oxylipin, (9Z, 11E)-13-oxooctadeca-9,11-dienoic acid, produced by the fungus *Penicillium herquei* isolated from the mangrove plant *Laguncularia racemosa*, showed minimal anti-parasitic activity against *Plasmodium falciparum* (half-maximal inhibitory concentration, IC_50_ > 100 µM), *Trypanosoma brucei* (IC_50_ > 100 µM), *Leishmania donovani* (IC_50_ > 100 µM) and *Leishmania major* (IC_50_ > 100 µM) [119]. Fortunately, other potentially bioactive compounds for *Trypanosoma brucei* have been investigated. Dihydroisocoumarins (trans and cis 4,8-dihydroxy-3-methylisochroman-1-one, 5-hydroxymellein and -mellein or 8-hydroxy-3-methylisochroman-1-one) and naphthoquinones (anhydrofusarubin, javanicin, dihydrojavanicin and solaniol) were produced by the fungi *Lasiodiplodia theobromae* and *Fusarium* sp. respectively, from the Malaysian mangrove plant *Avicennia lanata* (Table 2). These compounds showed significant activity against *Trypanosoma brucei brucei* with IC_50_ values of 0.32–12.5 μM [75].

It is worth noting that the bioprospection of bioactive compounds against protozoan must take into account their cytotoxicity for the protozoan host cells [120]. Among thirty-four active fungal extracts assayed against the amoeba *Naegleria fowleri,* by Demeres et al. [121], two were detected with high cytotoxicity on the J774 macrophage cell line (IC_50_ < 5 µg/mL). For *Leishmania donovani* infecting the same macrophage lineage, those authors found 562 extracts active at 10 µg/mL or less. However, when they established a criterium for high antiparasitic activity (IC_50_ < 1.0 µg/mL) and low cytotoxicity (J774 IC_50_ > 5 µg/mL), only 116 remained. Besides, 64% of these 116 extracts were found when the endophytic fungal strains were grown under the influence of epigenetic modulators [121]. By contrast, these cytotoxic compounds may be useful for cancer and tumor treatments [119,121,122,123].

The trypanosomatid *Trypanosoma cruzi* is the etiologic agent of Chagas disease. Although only about 10% of people infected are diagnosed, it has been estimated that 6–7 million people worldwide (endemic in Latin America) may be infected with *T. cruzi*, putting over 70 million people at risk [124]. In this sense, bioprospection of trypanocidal molecules is of undoubted interest to public health, mostly because Chagas disease is considered a neglected tropical disease, as pharmaceutical companies do not normally show interest in it. Fortunately, extracts of endophytic fungi have shown promising results, with high activity against trypanosomatids [87,121]. Ferreira [120] showed that extracts of the endophytic fungi Diaporthe cf. mayteni and Endomelanconiopsis endophytica have high trypanocidal activity against amastigote forms of *T. cruzi*. In another study of the same group, ophiobolin K and 6-epi-ophiobolin K (two bioactive compounds) were isolated from *Aspergillus calidoustus* and shown to be effective against this trypanosomatid [122]. However, despite being a promising approach to fight Chagas disease, there is still a lack of specific studies on mangrove-isolated endophytes against *T. cruzi*.

### 3.2. Antibacterial

Animal management is essential for the improvement of livestock. Nonetheless, this interaction led to the appearance of several foodborne diseases [125,126]. Diseases that can spread among several different animal species affecting are defined as zoonotic diseases. Among the groups of bacteria, zoonotic enterobacteria are highlighted for possessing resistance to pH and temperature variations [127]. One factor that influences the low efficiency of pesticides in agriculture is the large-scale administration of synthetic antibiotics to the healthcare of humans and animals, which also has a role in selecting plasmids of resistance to synthetic chemicals [128,129,130].

In this sense, the search for alternatives such as endophytic fungi bioactive compounds may overcome the bacterial resistance problem, and consequently mitigate the environmental impact caused by high amounts of inefficient pesticides. Aflatoxin B2b mycotoxin produced by *Aspergillus flavus* associated with Chinese mangrove plant *Hibiscus tiliaceus* (Table 2) showed antibacterial activity against *Escherichia coli, Bacillus subtilis* and *Enterobacter aerogenes*, with IC_50_ values of 22.5, 1.7 and 1.1 μM [40]. In addition, sesquiterpenoids compounds (Table 2), isolated from an *Aspergillus* spp. strain found at *Xylocarpus moluccensis,* presented moderate antibacterial activities against *Staphylococcus aureus,* with IC_50_ values from 31.5 to 41.9 μM [51].

Another compound was isolated from *Pestalotiopsis* sp. present in the leaves of *Rhizophora mucronata* and collected in the region of Dong Zhai Gang-Mangrove Garden on Hainan Island, China. This compound possesses a novel hybrid sesquiterpene-cyclo-paldic acid metabolite with an unusual carbon skeleton, called pestalotiopisorin A. Antibacterial activity was evaluated against *Enterococcus faecalis*, showing moderate results [91] (Table 2). The tetracyclic triterpenoids 12α-acetoxy-4,4-dimethyl-24-methylene-5α-cholesta-8-momoene-3β, 11β-diol, 12α-acetoxy-4,4-dimethyl-24-methylene-5α-cholesta-8,14-diene-2α,3β,11β-triol, and meroterpernoids Guignardone B, Guignardone I, Guignardone A and Guignardone J, isolated from the fungi *Guignardia* sp and *Phyllosticta capitalensis*, were also investigated for inhibitory activity against several bacteria, such as *S. aureus, E. coli, Micrococcus tetragenu* and *Pseudomonas aeruginosa* (Table 2) [131].

A new isocoumarin derivative (pestalotiopisorin B), isolated from the endophytic fungus *Pestalotiopsis* sp., was shown to be active against *P. aeruginosa*, *methicillin-resistant S. aureus, B. subtilis* and *E. coli*. The fungus was isolated from *Rhizophora stylosa*, a plant present in a mangrove area of China [94]. Numerous compounds were also obtained from the culture of *Ascomycota* sp. found on *Pluchea indica*, collected in Shankou Mangrove Nature Reserve in the Guangxi Province, China. The obtained compounds showed antibacterial activity against the Gram-positive *S. aureus* and *B. subtilis*, and the Gram-negative *E. coli*, *K. pneumoniae* and *Acinetobacter calcoaceticus*. These compounds were identified as dichloroisocoumarins–dichlorodiaportintone, desmethyldichlorodiaportin and dichlorodiaportin [83].

*Kandelia candel* is a plant spread in Guangdong province, China, a host of *Guignardia* sp., which produces Guignardins B and palmarumycin BG1. Among them, Guignardins B presented antibacterial activity against *E. faecalis* ATCC 29,212 and another one against *Aeromonas hydrophila* ATCC 7966 [131]. *Heritiera fomes* is a mangrove plant located in the region of Sundarbans, India. The endophyte isolated from it is *Pestalotia* spp., which produces oxysporone, a compound containing a 4H-furo(2,3-b)pyran-2(3H)-one structure, and xylitol, a molecule with five-carbon sugar alcohol. Both compounds demonstrated efficiency against *methicillin-resistant S. aureus* (MRSA) strains ATCC 25,923, RN4220, EMRSA-15, EMRSA-16, SA-1199B and XU2, with IC_50_ values ranging between 32 and 128 µg/mL [48].

### 3.3. Antiviral

The viruses are intracellular-dependent, and are always necessary for the invasion and kidnap of cellular machinery to replication. Enveloped viruses differ from non-enveloped ones in these situations. Enveloped viruses tend to fuse their membrane to release the genome inside the cytoplasm of the host using cytoplasmic endosomes. This way, fusogenic peptides that work in low pH facilitate access to cytoplasmic endosomes. In response, the release of molecules by the cells can prevent pH lowering, which inhibits the capability of virion fusion.

Non-enveloped viruses (such as enteroviruses) accumulate in endosomes and present high acidity. Identification of these viruses depends on the receptors exposed on the surface of the cells, to which viruses attach. Enteroviruses usually read α2β1 integrin, while adenoviruses and coxsackieviruses use coxsackie and adenovirus receptors [132,133]. Viruses with an RNA genome initiate their translation and transcription in the cytoplasm, turning them into specific targets to inhibitors inside the cell. On the other hand, DNA viruses need to penetrate the nucleus to start the process of replication. During translation and transcription, there is an abundance of proteins and viral polymerases, thus creating a target for drugs with inhibitory action. Assembly of non-enveloped viruses generally occurs in the cytoplasm, lysing the cell and spreading viral particles to other cells [134]. In addition, replication alters the functions of endosomes, as well as ER and Golgi, required for viral replication [135,136]. Even cholesterols and lipidic structures are unusual, making them targets for antiviral drugs [137,138].

Viruses essentially depend on manipulation of apoptosis to successfully replicate. This is necessary for the virus to interrupt anti-apoptotic growth factors in the early stages of replication and boost replication at the end of the cycle, with pro-apoptotic caspases assisting in viral dispersion in the cells. Considering this, some drugs exploit cellular apoptosis activity, which includes, for example, the recognition of viral invasion by pattern recognition receptors (PRRs) and the signaling to Bcl-2 proteins, proteins essential to the apoptosis process by regulation of pro-apoptotic and anti-apoptotic intracellular signals [139,140,141]. The process initially includes the recognition of viral invasion by pattern recognition receptors (PRRs) and the signaling to Bcl-2 proteins, proteins which are members of the B cell lymphoma 2 (BCL-2) gene family. 

The replication of the hepatitis C virus recruits a NS3 protease, which is a serine protease with activity at the amino-terminal and helicase function at the carboxyl-terminal. These two functions elect NS3 as an efficient target for antiviral therapy [142,143].

Paclitaxel is a compound initially isolated from parts of western yew in 1960, and is used as an anticancer drug [144]. Endophytic fungi were identified as feasible alternatives as producers when compared to western yew producers [145]. *Fusarium oxysporum* endophytic fungi isolated from *Rhizophora annamalayana* was observed as an example of a paclitaxel producer [146]. Besides having anticancer and antitumor activities, paclitaxel also presents anti-HIV activities, acting in the processes before and after viral invasion. At a concentration of 20 μg/mL, paclitaxel from fungi provided a 66% inhibition efficiency against the HIV-1 pseudo-virus. Although the inhibition effect over HIV-1 integrase has been relatively weak, the inhibition activity against the viral protease was as high as that observed with pepstatin A (a known HIV-1 protease inhibitor), which was enough to hinder the success of viral replication [147].

The *Neosartorya udagawae* HDN13-313 strain (which metabolome presents the Neosartoryadins A and B secondary metabolites) was found in roots of the mangrove plant Aricennia marina. These fumiquinazoline alkaloids displayed, respectively, IC_50_ values of 66 and 58 μM against the virus H1N1, which is a better result than that observed for the synthetic drug Ribavirin (IC_50_ = 94 μM) [76]. Other compounds investigated concerning H1N1 antiviral activity were the Emerimidine A and B isodolines and the pestalotiopsone F, pestalotiopsone B, 3,8-dihydroxy-6-methyl-9-oxo-9H-xanthene-1-carboxylate and 5-chloroisothiorin polyketides. The isodolines and polyketides were isolated from the *Emericella* sp. fungus of the mangrove plant *Aegiceras corniculatum* and the *Pestalotiopsis* spp. fungus of the mangrove plant *Rhizophora stylosa* [63] (Table 2). These studies demonstrate the value of biocompounds obtained from endophytic fungi as a source of a new, unexplored, bioactive niche of biocontrollers for pathogens such as protozoan, bacteria and viruses.

## 4. Future Challenges

The abundance of natural compounds present in mangrove areas is an example of biocompound richness, given the plethora of unexplored secondary metabolites [148]. Several studies have reported this production of pathogen biocontrollers as a defense mechanism developed to the presence of fungi in plants [149,150]. Beyond the natural benefits of exploring this niche, there are other ways to obtain such metabolites. In general, these molecules are produced in low quantities as part of the plant’s defense mechanisms. However, some techniques may enhance the production of secondary metabolites, such as strain improvement, one strain–many compounds (OSMAC), epigenetic modulation and conducted stress. 

A common technique used for this purpose is co-cultivation. It consists in cultivating microorganisms that are antagonistic or that depend on the same resources, which leads to competition. Several of these compounds are not produced in axenic cultivation. Nevertheless, co-cultivation makes it possible to stimulate cryptic compounds, allowing for the discovery of new molecules [151]. Co-cultivation of marine-derived fungi *Emericella* spp. and actinomycete *Salinispora arenicola* allowed for the discovery of compound Emericellamides A and B, which presented antibacterial activity [152]. Compounds such as Neoaspergillic acid, Ergosterol and Aspergicin were isolated utilizing co-cultivation of mangrove epiphyte and present antibacterial activity against Gram-positive bacteria [153]. 

The application of epigenetic elicitors presents a viable niche to be explored, albeit the omics knowledge and genome data are still unclear and require further studies. Beyond that, the knowledge hitherto obtained about evolution, ecology and interaction pattern with plants and other microbes is limited, hampering the discovery process. Another difficulty is the long process of screening strains and obtaining new compounds. In addition, the process of deciphering bioactive compounds from endophytes in lab conditions entails a diminution of metabolite production compared to the yield result of repeated subculturing.

## Figures and Tables

**Table 2:** Chemical class of the main anti-protozoan, antibacterial and antiviral metabolites produced by mangrove endophytic fungi.

Chemical Class	Compounds Isolated	Fungus	Host Plant(s)	Localization	Biological Target	Reference
**Isocoumarin** ** 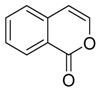 **	trans and cis 4,8-dihydroxy-3-methylisochroman-1-one, 5-hydroxymellein and -mellein or 8-hydroxy-3-methylisochroman-1-one	*Lasiodiplodia theobromae*	*Avicennia lanata*	Terengganu, Malaysia	*Trypanosoma brucei brucei*	[75]
**Naphthoquinones** ** 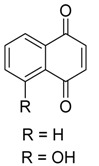 **	Anhydrofusarubin, javanicin, dihydrojavanicin and solaniol	*Fusarium* sp.	*Avicennia lanata*	Terengganu, Malaysia	*Trypanosoma brucei brucei*	[75]
**Aflotoxin-derived mycotoxin** ** 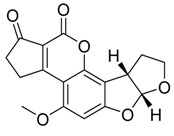 **	Aflatoxin B2b	*Aspergillus flavus*	*Hibiscus tiliaceus*	Hainan province, China	*E. coli*, *B.* *subtilis* and *Enterobacter aerogenes*	[40]
**Sesquiterpene** ** 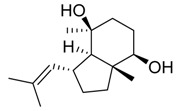 **	(7 S, 10 S)-7,10-ácido epoxysydonic; (7 R, 11 S) -7,12-epoxi s ácido ydonic; ácido 7-desoxi-7,14-didesidro-12-hydroxysydonic; (E) -7-desoxi-7,8-didesidro-12-ácido hydroxysydonic Pestalotiopen A	*Aspergillus* sp.	*Xylocarpus moluccensis*	Trang Province, Thailand	*Staphylococcus aureus*	[40,51]
		*Pestalotiopsis* sp.	Rhizophora mucronata	Hainan Island, China	*Enterococcus faecalis*	[91]
**Polyketide-derived mycotoxin** ** 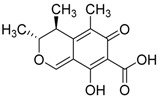 **	12α- acetoxy- 4,4-dimethyl-24-methylene-5α-cholesta-8-momoene-3β,11β-diol, 2α-acetoxy-4,4-dimethyl-24-methylene-5α-cholesta-8,14-diene-2α,3β,11β-triol	*Penicillium* sp.	*Bruguiera sexangula* var. *Rhynchopetala*	China	*S. aureus, E. coli* and *Micrococcus tetragenu*	[88]
	Guignardone B and Guignardone I	*Guignardia* sp.	*Scyphiphora hydrophyllacea*	Hainan Province, China	*Staphylococcus aureus* (MRSA) and *S. aureus*.	[71]
	Guignardone A and guignardone J	*Phyllosticta capitalensis*	*Bruguiera sexangula*	Southern China	*P. aeraeruginosa* and *S. aureus*	[92]
**Coumarin** ** 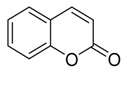 **	Austinol	*Penicillium* *citrinum*	*Bruguiera* *sexangula var.* *rhynchopetala*	South China	*S. aureus* *S. epidermidis*	[85]
	Bacillisporin A), bacillisporin B and Penicisimpins A–C	*Penicillium simplicissimum*		Hainan Island, China	*Bacillus subtilis, Aeromonas hydrophilia*, *Escherichia coli*, *M. luteus*, *Pseudomonas aeruginosa, V. alginolyticus, V. harveyi* and *V. parahaemolyticus*	[88]
**Isocoumarin** ** 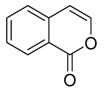 **	Dichlorodiaportintone, desmethyldichlorodiaportin, dichlorodiaportin	*Ascomycota* sp.	*Pluchea indica*	Guangxi Province, China	*S. aureus, B. subtilis, E. coli, Klebsiella pneumoniae* and *Acinetobacter calcoaceticus*	[37]
	Spergillumarinas A and B	*Aspergillus sp.*	*Bruguiera gymnorrhiza*	SouthChina	*S. aureus* and *B. subtilis*	[49]
	Penicimarins G and H	*Penicillium citrinum*	*Bruguiera sexangula var. rhynchopetala*		*S. aureus,S. epidermidis*, *Escherichia coli, Bacillus cereus* and *Vibrio alginolyticus*	[85]
	Pestalotiopisorin B	*Pestalotiopsis sp.*	*Rhizophora stylosa*	Hainan Island, China	*E. coli* and *P. aeruginosa*	[93]
**4H-chromen-4-one** ** 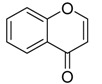 **	8-dihydroxy-chromone, bacillisporin A and bacillisporin B	*Penicillium aculeatum*	*Kandelia candel*	Yangjiang, Guangdong province, China	*B. subtilis* and *Salmonella* spp.	[94]
**Xanthones** ** 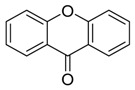 **	3,6,8-trihydroxy-1-methylxanthone	*Nigrospora* sp.	*Pongamia pinnata*	China	*MRSA, E. coli* and *S. epidermidis*	[117]
**Anthraquinone** ** 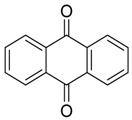 **	Isoversicolorin C, versicolorin C	*Aspergillus* *nidulans*	*Rhizophora stylosa*	Twig, Chanthaburi Province, Eastern Thailand	*E. coli, M. luteus, V. vulnificus, V. anguillarum,* *V. alginolyticus, Ed.* *ictaluri,* *V. parahaemolyticus* *S. aureus* and *E. Faecalis*	[45]
	Diaportheins B and Emodin	*Eurotium chevalier*	*Rhizophora* *Mucronata*	Hainan Island, China	*E. coli*	[67]
	9-dehydroxyeurotinone	*Eurotium rubrum*	*Hibiscus tiliaceu*	South China	*Staphylococcus aureus* and *Escherichia coli*	[68]
	*Bostrycin, and Deoxybostrycin*	*Nigrospora* sp.	*Kandelia candel*		*S.aureus, E.coli, P. aeruginosa, Sarcina ventriculi, B. subtilis*	[78]
	2′-acetoxy-7-chlorocitreorosein	*Penicillium citrinum*	*Bruguiera sexangula* var. *rhynchopetala*		*Vibrio parahaemolyticus*	[84]
	2R,3S)-7-ethyl-1,2,3,4-tetrahydro-2,3,8-trihydroxy-6-methoxy-3-methyl-9,10-anthracenedione	*Phomopsis* sp.	*Rhizophora apiculata*	Songkhla province, Thailand	*Staphylococcus aureus ATCC25923* and *methicillin-resistant S. aureus SK1*	[96]
	2-O-acetylaltersolanol B, Altersolanols A and B	*Stemphylium sp.*	*Bruguiera sexangula var. rhynchopetala*	South China	*E. coli, S. aureus* and *B.* *subtilis*	[96]
**Naphthoquinones** ** 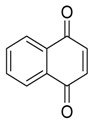 **	5-hydroxy-2-methoxy-6,7-dimethyl-1,4-naphthoquinone	*Daldinia eschscholtzii*	*Bruguiera sexangula var. rhynchopetala*	South China	*B. cereus*	[62]
	6-hydroxy-astropaquinone B, astropaquinone D and 3-O-methyl-9-O-methylfusarubin	*Fusarium* *napiforme*	*Rhizophora mucronata*	South Sulawesi Province, Indonesia	*S. aureus* and *P.aeruginosa*	[69]
**Biphenyl** ** 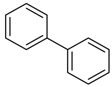 **	5,50-dimethoxybiphenyl-2,20-diol	*Phomopsis longicolla*	*Brguiera* *sexangula var.* *rhynchopetala*	South China	*Vibrio parahaemolyticus*	[38]
***N*** **-phenylnaphthalen-1-amine** ** 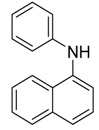 **	Nigronapthaphenyl	*Nigrospora sphaerica*	*Bruguiera gymnorrhyza*	City of Galle, Sri Lanka	*B, subtilis* and *Bacillus cereus*	[115]
**Alkaloids** ** 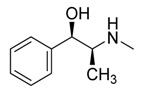 **	GKK1032C	*Penicillium* sp.	Mangrove plant Chinese	Hainan province, China	*methicillin-resistant S. aureus*	[90]
	Penicibrocazines B–E, Bbrocapyrrozins A and 4-hydroxy-3-phenyl-1H-pyrrol-2(5H)-one	*Penicillium brocae*	*Avicennia marina*	China	*S. aureus*, *Micrococcus luteus*	[80,81]
	Socromen-1-ona and 3, Ácido 4-dihidroxibenzóico	*Phyllosticta capitalensis*	*Bruguiera sexangula*	Southern China	*P. aeruginosa, S. aureus, B. subtilis* and *E. coli*	[92]
**Sesquiterpene** ** 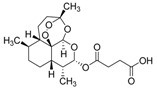 **	Infectopyrones A and B	*Stemphylium* sp.	*Bruguiera sexangula* var. *rhynchopetala*	South China	*B. subtilis Micrococcus tetragenus*, *Micrococcus luteus* and *S. albus*	[101]
***p*** **-quinone macrolactam** ** 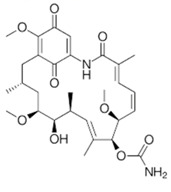 **	Cytosporone E	*Acremonium* *Strictum*	*Rhizophora apiculata*	Island of CatBa, Vietnam	*S. aureus*	[32]
	Ent-cladospolide F	*Cladosporium cladosporioides*	*Bruguiera gymnorrhiz*	Hainan Island, China	*S. aureus*	[118]
	(2S)-2,3-dihydro-5,6-dihydroxy-2-methyl-4H-1-benzopyran-4-one and 4-ethyl-3-hydroxy-6-propenyl-2H-pyran-2-one	*Colletotrichum gloeosporioides*	*Ceriops tagal*	Hainan Province, China	*Micrococcus tetragenus, S. aureus, Streptomyces albus, B. cereus* and *B. subtilis*	[60]
	Cytospomarin	*Cytospora* sp.	*Ceriops tagal*	Hainan Island, China	*E. coli* and *M. oryzae*	[61]
	8-O-methylnodulisporin F and nodulisporin H	*Daldinia eschscholtzii*	*Brguiera sexangula var. rhynchopetala*	South China	*Staphylococcus aureus, methicillin-resistant S.* *aureus (MRSA)* and *Bacillus cereus*	[62]
**Antiviral compounds**						
**Isoindolone** ** 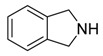 **	Emerimidine A and B	*Emericella* sp.	*Aegiceras corniculatum*	HaiKou, China	H1N1	[65]
**Alkaloid** ** 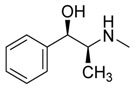 **	Neosartoryadins A and B	*Neosartorya hiratsukae*	*Aricennia marina*	China	H1N1	[78]
***p*** **-quinone macrolactam** ** 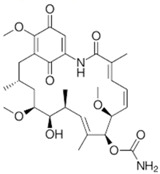 **		*Diaporthe* sp.	*Rhizophora stylosa*	Hainan Province, China	H1N1 and H3N2	[63]
	pestalotiopsone F, pestalotiopsone B,3,8-dihydroxy-6-methyl-9- oxo-9H-xanthene-1-carboxylate, and 5-chloroisorotiorin	*Pestalotiopsis* *vacinii*	*Kandelia candel*		Anti-enterovirus 71 (EV71)	[116]

## Data Availability

The data presented in this study are available upon request from the corresponding authors.
